# Hospital and Institutional News

**Published:** 1912-04-27

**Authors:** 


### HOSPITAL AND INSTITUTIONAL NEWS.

### THE "TITANIC" DISASTER AND AFTER.

The general public has expressed its sympathy with the sufferers in the loss of the "Titanic" so prominently by subscribing to the funds which the Lord Mayor, Sir Thomas Crosby, M.D., and the various daily newspapers have opened, that we think it only accurate to remind our readers that the institutional world is also playing its part in this respect. Hospitals for the sick, except those in New York, which would receive passengers and crew in the ordinary routine of work, lie naturally beyond the sphere of action, but not so institutions for the permanently disabled or the permanently deserted and bereaved. Thus Dr. Barnardo's Homes, and more than one orphanage, have offered to admit either a specific number, or, as in the case of the former, any number of destitute children who have been made orphans by the disaster. Southampton has suffered more than any other town, and therefore we hope that Southampton institutions will be especially open handed in this matter.

### AN ENGLISH HOSPITAL ON THE RIVIERA.

The recently unveiled memorials to Queen Victoria and the late King, which France has erected on the Riviera, attract added attention to the work of the English hospitals in the South of France. The Queen Victoria Hospital at Nice is especially connected with hospital life at home, for its President is Sir George White, so well known in Bristol, and beyond it, as the President of the Royal Infirmary. At the Nice Hospital nearly half the patients are free, and one-tenth pays five francs per day or less. This shows that the hospital is fulfilling its purpose, which we may remind our readers is to minister to English and Americans who may find themselves in straits, as Sir George White put it, and in need of hospital care. Many private patients also outside the hospital rely upon it for nursing. As twelve per cent. of the patients are Americans, it will be seen that the hospital does an important work for the English-speaking population.

### A DISCUSSION ON THE ALMONER'S WORK.

Owing to the large number of patients interviewed by Miss Gregory, lady almoner to the West Ham and Eastern General Hospital, a discussion has arisen as to the best method of supervising her work. Miss Gregory has pointed out that the large number of cases, between 400 and 500 within five weeks, precludes the possibility of reporting on each case separately. One committeeman said that only by giving some report could the lady almoner satisfy those who were opposed to the appointment. Another said that in not filling up her report book she had not obeyed instructions. It was then pointed out that the King's Fund would only give grants to hospitals which appointed almoners, and on the suggestion that, backed by a committee, the almoner could save the hospital a large amount of money it was decided to appoint a committee "to deal with the almoner's report." From the reports of the discussion it is not very clear what precisely this committee's instructions were, but it is evident from the statement of the Chairman the almoner "has never been dealt with in any consistent way since her appointment," that the blame lies with want of system rather than with its nominal head. The number of cases mentioned works out at 90 per week, or 18 per day, excluding Saturdays and Sundays. To visit all of these and report on all would appear more than can be expected of one unassisted individual, though no doubt no case is visited without some notes being made. For a rough-and-ready system the Hospital Almoners' Council should be applied to, but it must be first of all admitted that on their first appointment almoners are often expected to do too much. The right way is at first to attempt to deal with no more cases than can be dealt with efficiently. Then when experience and a system begins to emerge to expand the system to wider limits.

### ST. MARY'S HOSPITAL AND SOLICITORS' TOUTS.

We are glad to follow up Mr. Reginald Garratt's letter in our columns last week with reference to the "Legal Aid" Society in the neighbourhood of the Royal Free Hospital, by noting what the current number of *St. Mary's Hospital Gazette* has to say with reference to the methods by which solicitors' touts are said to procure the names and addresses of St. Mary's out-patients. "Either," says the *Gazette*, "the agent buttonholes a patient in the casualty or out-patient department, or, possibly with the aid of a third person, the agent has access to the books of the hospital." Dismissing the first alternative as improbable, the writer offers the "unpleasant possibility that some one employed in the hospital . . . is in league with a solicitor or with his agent." The suggested remedy is that "the casualty books be fitted with locks, and that none but the casualty House Surgeon and the Resident Dresser have a key." We confess to taking a kinder view of St. Mary's staff than the *Gazette* feels impelled to do, and the remedy suggested seems inconvenient in view of the number of times in the day when the casualty books have to be consulted. This would surely place on the casualty House Surgeon an additional responsibility which the precaution suggested would not help him to fulfil, and, in short, add another worry to his harassed existence. Is it not likely that at St. Mary's, as at the Royal Free, Mr. Garratt is right in stating that "the information that these societies obtain can only be through persons after leaving the hospital, or whom they may have observed to be connected with patients brought in"?

### AN AMERICAN HOSPITAL REFORMER.

The founder of the American Red Cross Society, Miss Clara Barton, died recently at the great age of ninety-one. She first became associated with hospital work by founding in Washington a bureau to organise the search of missing men for whom inquiries were made at the end of the American Civil War. By its means, according to the *Times*, the graves of no fewer than 12,000 soldiers are said to have been identified. But apart from this Miss Barton was known to Europe for assisting in organising hospitals during the Franco-Prussian War, and her services were rewarded by the Iron Cross with which she was decorated by the German Emperor. It was after her return from Europe in 1873 that she began her attempt to found a branch of the Red Cross Society and to include America in the Treaty of Geneva. At the end of eight years she was successful. She also introduced an American amendment to the scope of the Red Cross, by which the Society should not confine its administration of relief to time of war, but should undertake it during national calamities. This amendment led Miss Barton to organise relief on several occasions between 1887 and 1900. She remained President of the Society until 1904.

### AN OLD HOSPITAL CUSTOM.

According to ancient custom the governors of the five Royal Hospitals—St. Batholomew's, St. Thomas's, Bridewell, Bethlem, and Christ's—in civic state attended Divine service at Christ Church, Newgate Street, on Wednesday last week, to hear the Spital sermon, which is preached annually about Easter. The service was at half-past two, and the preacher the Bishop of Bristol. Sir Thomas Crosby, M.D., as Lord Mayor, with aldermen, sheriffs, and corporation, was also present. In his sermon the Bishop of Bristol drew a parallel between the latest discoveries of science and the aims of religious teachers, showing that what science achieved in the world of natural law, religion aimed at achieving in the world of spiritual experience. The service is of interest in a special sense to-day as a reminder to all the clergy and to those who work for the cause of Hospital Sunday, that the pulpit has from the earliest times been the ally of those who care for the sick.

### TWO NEEDS AT WORCESTER INFIRMARY.

Attention has been called at the Worcester General Infirmary to the large sum of £125 which has been spent on water for the year 1911, the amount used daily showing an average of nearly 100 gallons per head. In this amount was included the water used in the laundry. The matter was much discussed and finally referred to the Finance Committee for investigation. It is hoped that the City Council will see its way to supplying the infirmary at a reduced rate. Another need has also been brought to light. The honorary medical staff has asked for a new *x*-ray installation, with a recommendation that a sum not exceeding £300 be spent on it. They urged that there could be no doubt as to the need of such a provision for the infirmary, which is said to be less well equipped than the hospitals at Hereford and Kidderminster in this respect. The committee has decided that it is their duty to provide the installation. It will be interesting to have the report of the Finance Committee as to the source of the waste of water complained of.

### THE COMMITTEE ON INDUSTRIAL DISEASES.

The medical members, who bear a proportion of two to five on the committee appointed by the Home Secretary to report whether cowpox, Dupuytren's contraction, and spasm of the eyelids, apart from nystagmus, can properly be added to those enumerated in the third schedule of the Workmen's Compensation Act, 1906, are Sir Clifford Allbutt, K.C.B., M.D., Regius Professor of Physic at Cambridge, and Dr. Thomas Morison Legge, M.D., Medical Inspector of Factories.

### THE PATENT MEDICINE INQUIRY.

Of the fifteen members of the promised Select Committee on Patent Medicines, the names of only a few have been mentioned up to the present. It is said that the Chairman will be Sir Henry Norman, who is member of Parliament for Blackburn; that the Labour Party will be represented by Mr. Hodge, and that Mr. Arthur Lynch, Mr. J. P. Hayden, Mr. W. S. Glyn-Jones, Mr. Harry Lawson, and Mr. C. Bathurst will be among the other members. Mr. Lynch, who is a registered medical practitioner and a man of many interests, has taken a great interest in the events which led up to the promise of the inquiry, and has frequently drawn the attention of the House of Commons to the vastness of the traffic in secret remedies. Mr. Glyn-Jones, the member for Stepney, is Parliamentary Secretary of the Pharmaceutical Society. There is reason to believe that the Committee will be of a thoroughly representative character.

### AN EFFECT OF THE COAL STRIKE AT CHICHESTER.

It is not at first apparent that a hospital report might be delayed because of the recent coal strike, but this has been the case at Chichester, where the West Sussex, East Hants, and Chichester General Infirmary has been unable to publish its annual report till quite recently, since the paper-mills which supplied the printers had been stopped on account of the coal strike. The report, we notice, has managed to attract local advertisers. The financial improvement in the infirmary's position and the credit due to Mr. Tom B. Harrison we have previously noted. The most important point apart from this is the King Edward VII. Reconstruction Scheme, which is making slow progress, though we learn that "an expert in administrative arrangement" is now working with the architect. Since the scheme has been much pruned down from its original scope, which we criticised in these columns at the time, we withhold further comment till the result of the joint deliberations of the infirmary's advisers is known. But, generally speaking, this hospital appears to have aroused itself of late to a very healthy condition of activity, the details of which are apparent by a comparison of its two latest reports.

### A MISER'S GIFT TO VIENNA.

Recent big bequests to hospitals in this country are more than equalled by the enormous sum of £100,000, which has been left to the Jewish community in Vienna to found a new children's hospital. One or two points about this bequest are worth noticing. The first is that the sum left comprised the whole fortune of the donor, an unusual fact, especially with bequests of this size. The second is that, although the medical and general staffs are to be appointed by trustees selected by the Jewish community's representatives, which thus become indirectly responsible for the hospital's management, the hospital itself will be thrown open to children of other religions. It should be added that the capital will not be available till various life annuities cease. Judging by comments in the *Jewish Chronicle*, the donor himself, Herr Joseph Spitzberger, was a man whose so-called modesty of life was only another name for parsimony of the extremest kind. He is stated to have lived in a garret, to have had a predilection for stale bread, to have saved the expense of taking a newspaper by consulting the news-sheets posted outside the offices of different journals, always to have gone afoot, and to have been his own laundress. Apart from such economies, his income was increased by successful speculating.

### THE MILNE TREATMENT IN ISOLATION HOSPITALS.

Although the profession at large has not given its unqualified approbation to that method of treating scarlet fever which may conveniently be referred to as the Milne treatment, it has nevertheless admitted that this method can claim considerable advantages under certain conditions. Where, for instance, it is deemed feasible to treat a case of scarlet fever at home, the method of Dr. Milne may enable the medical attendant to follow the case throughout with but small risk to the other occupants of the house. But even then it is necessary that a very competent nurse should also be engaged. As regards the employment of this treatment in fever hospital wards, there is nothing to be gained. What are known as septic cases are already isolated in some way or another, and nowadays the duration of the residence of scarlet-fever patients is considerably shorter than it used to be. Nevertheless the Milne treatment continues to attract the attention of local authorities, some of whom see in it a method which makes strongly for economy and simplicity. Several boards having control of isolation-hospital accommodation have asked their medical officers to report on the matter before involving themselves in large schemes of expenditure for additional pavilions. We had recently in these columns a reference to the case of Dr. Butchart, who was practically dismissed from his appointment as medical superintendent of a fever hospital in Scotland for the reason that he refused to experiment on the patients committed to his care with this very same method of Dr. Milne. Owing to the support given to Dr. Butchart by the medical Press the authority in question will have very great difficulty, we believe, in obtaining the services of an efficient medical superintendent. We note that, in his recently issued annual report, Dr. Robertson, Medical Officer of Health, for Leith, is also of opinion that for hospital cases at any rate there is nothing to be gained by adopting the strict Milne treatment.

### RESEARCH IN FEVER HOSPITALS.

In general terms it is true to say that our knowledge of the infectious fevers has not progressed during the last two decades so markedly as has that of certain other branches and specialties of medicine. We are not unmindful of the crushing defeat which von Bering administered to diphtheria by the discovery of anti-diphtheritic serum; and in diagnosis the Widal reaction, Koplik's spots, and other aids to certainty have proved their worth. But most of the methods devised for preventing, treating, or diagnosing fevers have proved to be comparatively disappointing, with a few brilliant exceptions to relieve the monotony. Why this should be is not easy to say. The medical officers of the fever hospitals are a keen, able, and instructed set of men. They have extraordinary facilities for studying the handful of diseases which alone are accepted in fever hospitals. Yet the amount of research which emanates from these clinics is distinctly meagre. In another column is printed a communication from a fever medical officer on the subject of Russo's reaction for typhoid fever, and the profession could do with more work of this kind from those who have specialised in that branch of practice. In this particular case it will be seen that the result of the research has been to show that Russo's test is of very little practical value, so far as the diagnosis of typhoid fever is concerned. Dr. Rankin holds that "it is of some diagnostic value"; but a study of his paper reveals so many exceptions, both positively and negatively, that for the ordinary general practitioner the test, simple as it is, seems hardly worth the trouble of performing. This, however, is a side issue which does not affect the point we are urging. The bacteriology, epidemiology, diagnosis, prophylaxis, and treatment of infectious fevers offer a field for original research the like of which probably does not exist elsewhere in medicine.

### "PEACEFUL PICKETING" IN THE PROFESSION.

A special meeting of the Bristol Health Committee for the appointment of a lady medical inspector proved abortive from the fact that the applicants, owing to a warning notice, declined to accept the appointment without the sanction of the British Medical Association, which had protested against the amount of work required at the salary offered. It happened that one candidate had come from Cambridge, not having seen the warning notice, and it was not until she was handed a notice by a clerk in the office that she was aware of the position. This attraction of the candidates' attention was resented by one member of the Health Committee. Mr. H. Anstey, who said that he was more convinced than ever before of the desirability of putting an end to peaceful picketing. Finally the question of the appointment was deferred, it being alleged that the Association had made a mistake with regard to the hours. The situation was not without its humour, but it is not easy for any professional organisation to find out who are candidates for any post except by attending on the day of application.

### A HOSPITAL SITE FOR LONDON UNIVERSITY.

Among the voluntary charities of London there is perhaps none more interesting to our foreign and provincial visitors than the Foundling Hospital, though Londoners themselves have a habit of taking it for granted, much as they do the National Gallery and the Tower of London. It is, therefore, with some mild degree of shock that the proposal will be contemplated for the acquisition of the extensive site of this institution as a building plot for London University. It is plausibly argued that the centre of London is by no means an ideal situation for a nursery, and that a removal to the country (as in the case of the Duke of York's School) would be in the best interests of all concerned. Certainly with the purchase money for the extensive site a fine new Foundling Hospital could be erected outside London in some more salubrious neighbourhood than that of Guilford Street; and London University could in turn be both better and more cheaply housed than on the alternative plot offered by the Duke of Bedford for a large sum of money. The main objection to be urged is against the consumption of one of the largest open spaces in that congested part of the metropolis: London has not so many of them that she can afford to let one of them be obliterated without due cause shown. There remains the point of view of the Governors of the Foundling Hospital, who may be loth to give up their historic halls.

### THE WINSLEY SANATORIUM EXTENSION SCHEME.

The latest report of the Winsley Sanatorium contains some interesting references to the extension scheme which was decided upon last October. The effect of this decision will be to raise the number of beds to 100. Mr. W. S. Skinner, of Bristol, is the architect, and his plans provide a new administrative block containing a self-contained house for the resident medical officer, the secretary's office and rooms for nurses forming the remainder of the building. This will set free nine rooms in the main building which is used for women patients, who will have a further eight rooms added, while twelve more beds for men will be provided in chalets or similar quarters. These additions necessarily involve important administrative changes which need not be discussed here. Suffice it to say that in all thirty beds will be added, and the board has decided to approach public bodies which may wish to accept the *pro rata* distribution. The scheme is expected to cost £9,000, of which about half is in hand.

### AN ATTRACTIVE HOSPITAL REPORT.

A very attractively got-up report has just reached us from the Royal Buckinghamshire Hospital, Aylesbury. A striking increase in the amount received from paying patients is recorded, together with a summary of the proceedings on December 12, which The Hospital then noticed, on the visit of Princess Victoria of Schleswig-Holstein, which was made an occasion for rallying the Berks, Bucks, and Oxon Branch of the League of Mercy. A footnote to the report refers to the resignation of the Rev. A. Smith, who has been chairman of the board for nine years, and a member of it for a very considerable period. The illustrations to the report leave nothing to be desired, and the lists of annual subscribers and donors are treated in such a way as not to overload the report with too extensive appendices of names. The secretary, Mr. Stanley E. Wilkins, may be congratulated upon the inviting appearance which he has succeeded in giving to this document, which even to-day is not always improved as much as it can be without undue extravagance.

### A NOVEL STATISTICAL RETURN.

A Local Government Board return of a novel nature has just been issued showing the incidence of infectious diseases during the past year in a manner which has hitherto not been possible. This improvement is due to an order issued a year and a half ago by the Board, making it incumbent on medical officers of health to transmit weekly to Whitehall statistics of the infectious diseases notified to them. A full analysis of the exact distribution of all the infective fevers, both as to season and locality, thus becomes for the first time feasible; and the report in question deals exhaustively with the three most serious notifiable zymotics—scarlatina, diphtheria, and enteric fever. The total number of notified cases of the first reached six figures, 104,617; of diphtheria there were 47,347 cases, and of enteric fever 13,730. The toll of child life and health taken by the first two of these is truly terrific, though the havoc wrought is less than it used to be. Of small-pox and typhus fever it is satisfactory to note that only 265 and 65 cases respectively were diagnosed; and no case of cholera occurred. The notification of pulmonary tuberculosis was not compulsory until November 1911, and therefore the returns must be far below what must be expected when the figures for this present year become available. But in spite of that more than 35,000 cases were notified.

### AN OLD CITY CHARITY.

The City of London Lying-in Hospital is not, perhaps, so well known to the public and the institutional worker as some of the larger institutions, such as those in Marylebone Road, York Road, and Endell Street. But it is a very old-established City charity which has done and is doing most valuable work in the City Road; and it takes care to keep abreast with the times. About four or five years ago a good deal of money had to be spent on bricks and mortar; and now with the expansion of its work it has been found necessary to resort to a festival dinner as a means of making both ends meet. This was celebrated last Monday at the Savoy Hotel, and the very satisfactory sum of £3,222 was realised. As a result of a recent appeal by the Lord Mayor, who was unavoidably absent from the dinner itself,

Mr. Albert Lewis has given £1,000 to name a ward in memory of his late wife. The hospital, it appears from the speech of the Chairman of the dinner (Mr. Alderman and Sheriff Hanson), was instituted in 1750, and has cared for 135,000 babies since that date. He also announced with regret the resignations of Mr. R. A. Owthwaite, the secretary, and Miss Fox, the matron.

### THE FEVER HOSPITAL CHAPLAIN.

We understand that the Rev. A. C. Wolston, chaplain at the South Eastern Fever Hospital, has been appointed, in addition, acting chaplain to the Brook Fever Hospital, Shooter's Hill, Woolwich, during the extension of sick leave which has been granted to the Rev. A. A. Dauncey.

### A NEW DISPENSARY FOR BRISTOL.

Though it began work two months ago, the Bristol Dispensary for the Prevention of Consumption was opened formally by the Lord Mayor last week. At the function in the Parish Hall Dr. Colston Wintle, Chairman of the Health Committee, presided. Owing to a gift of £800, through the Bristol Civic League, the dispensary was started, and it has since received a present of tuberculin from Switzerland. The medical officer in charge is Dr. C. J. Campbell Faill. The Chairman drew attention to the example which Edinburgh had set in the matter of such dispensaries. The object of the Bristol institution is to reach those in contact with consumptives. Since it has started work nearly fifty incipient cases have been discovered. Dr. D. S. Davies, Medical Officer of Health, said that the dispensary dovetailed with the Health Department, and Dr. Faill recalled that already 241 cases had been dealt with. All are sent by practitioners, hospitals, or the Medical Officer of Health. Family histories are made up as far as possible. It is much to be hoped that what may be called the Edinburgh system, which has been described before in our columns, will develop to a similar extent in Bristol. Dr. Philip, of Edinburgh, telegraphed his good wishes. We may add that the building itself is very simple. The ground floor is given up to a waiting-room and to an office, and above are the consulting and dressing rooms and the laboratory.

### QUESTION OF A REDECORATED WARD.

More than one speaker at the annual meeting of the Gravesend Hospital last week alluded to the comments which we have made on the need for redecorating the wards in the north block. There is no need to repeat these comments here, but they were aptly timed, for the discussion which they provoked at the annual meeting should, if properly managed, lead to further public interest, and such interest gives the first chance of securing the necessary money. It is indeed as a stimulus to such interest that annual meetings have their real value, and if any of the public with experience in the ordinary routine of annual meetings doubts this, that is because the annual meeting nowadays is often a purely perfunctory affair. It should not, and as Leicester Infirmary shows each year, it need not be so. We are especially glad, therefore, that the Gravesend annual meeting this year managed to focus itself upon the need for the redecorated ward for which we have argued. Mr. H. D. Stephenson we believe to be too good a chairman of committee to let the matter rest here. In a frankly amusing speech he gave the reasons why this improvement has been delayed for two years. Can he not with equal cogency find reasons for pleading a cause that never ceased to inspire Florence Nightingale? The matter really should not be allowed to slide any longer, and as experience has taught us that money can always be found, if sufficient energy is displayed in attempting to raise it, we shall be glad to give publicity to any scheme or appeal which Mr. Stephenson may launch. The present revived interest in this improvement must not be allowed to wane without result.

### HOSPITALS AND PAYING PUPILS.

A novel suggestion for raising money for the hospitals, and for spreading an elementary knowledge of hygiene and sick management among women of the middle classes, has been put forward in the lay Press. The writer suggests that the hospitals should receive paying pupils. These, it is explained, must not be confounded with paying probationers, because the object of the instruction would be elementary teaching for use at home, and not in any sense a professional training. This indeed, is obvious, from the fact that such pupils would be received only for the short period of a month, or a little longer. The hospital staff, it is added, should teach and not merely leave the pupils to acquire knowledge, while the pupils' duties should be solely confined to the patient, and should not include "dusting or sweeping, which can be practised in any household." Objections to the scheme are very obvious. The first is that the work of a hospital is done at such a high pressure, and under such discipline, that a continual migratory stream of lay helpers totally ignorant of hospital work and attempting to learn in one month what must be learnt professionally if it is to be learnt at all, would threaten to disorganise all procedure. The elementary duties objected to are as much insisted on to accustom probationers to the hospital atmosphere as for their own sake. No fees, we fear, which such middle-class women could afford to pay could compensate the hospitals in any degree for the inconvenience and indiscipline which their proposed invasion might cause. The experience of the London Hospital would be interesting, as a scheme has been tried there recently on somewhat similar lines.

### OVERCROWDING A MENTAL HOSPITAL.

The nominal accommodation of Denbigh Mental Hospital is for 917, but the clerk, Mr. W. Barker, has stated at the yearly meeting of visitors and subscribers that practically 1,000 patients could be provided for. At the end of March there were apparently 971 patients in the institution. More than one speaker urged that the attention of the Commissioners should be called to the matter,

Mr. R. I. Hughes being particularly emphatic on the point. Eventually it was decided to inquire what the Government was going to do in regard to the care and treatment of the feeble-minded. This, of course, is only one illustration of a common condition of affairs, but it is a peculiarly apt one at the present time, when Dr. Robert Jones has brought the whole matter into prominence. The first thing, of course, is to relieve the overcrowded institutions, and every example of the need for Government action that can be cited must be brought forward as additional evidence. At Denbigh the re-elected chairman, Mr. J. A. Chadwick, said that the committee would have to concentrate on providing extra accommodation. If this means to squeeze more patients into already overcrowded buildings, we must disagree, but the proposal is only the latest indication of an increasingly pressing difficulty.

### A DIFFICULT POSITION AT NORWICH.

The operation block, which has been nearing completion at the Norfolk and Norwich Hospital, was sufficiently ready to be inspected by those who attended the recent annual meeting. Two theatre units have been built on the site of the original theatre, which was constructed thirty years ago. But it was not exclusively to these that Mr. R. E. Horsfall, Chairman of the Board, directed the attention of those who attended. He had to enlarge upon the year's debt of £2,385, and to admit the total debt to be nearly £8,000. In view of our recent series of housekeeping articles, it is interesting to add that Mr. Horsfall attributed the decrease per head in the cost of provisions to be due to the lady superintendent, which is all the more striking an illustration of the points for which our articles contended, since there has been a general rise in the price of provisions. Mr. Horsfall thought the hospital's financial outlook worse than it had ever been, and attributed present difficulties to the number of relatively well-to-do residents who contributed nothing to the institution. The Master of the Rolls, who presided, gave a well-deserved tribute to the ability of the speakers, which made the meeting a very interesting one, though we agree with him in thinking that Mr. Horsfall had understated his case in complaining of the small support gained from places of worship.

### ARE LEGACIES TO HOSPITALS DECLINING?

The Chairman of the Finance Committee of the Norfolk and Norwich Hospital, Mr. Charles Larking, has given some interesting figures of the legacies bequeathed to other hospitals during the past twelve months, and in the light of them courageously refuses to believe that the charitable are being deterred from giving. He suggested following the example of St. Bartholomew's and appointing a special committee of governors, who were not on the executive, to help in raising funds. The hospital would not benefit one farthing from its compulsory payment, of some £20, under the Insurance Act. We have frequently contended that his is the right view, and if it remains so after the Insurance Act has been a year or two in operation, the voluntary system will be able to claim at least nine lives.

### DEFORMING THE FEET IN ENGLAND AND CHINA.

The folly displayed by the human race over the selection of footgear seems to be never ending. It is true that the Chinese are rapidly abandoning the custom of crippling and deforming the feet of the upper-class women from infancy onwards; but then the Chinese are an intelligent people, and willing to listen to the voice of reason. In this country no such sensibility prevails; and there is good evidence that the infection is spreading to the working classes from those who ought to set them a better example. The pointed toe, prolific cause of bunions, corns, hammer toes, flat feet, and other painful deformities, seems to have obtained a rooted hold upon the educated classes, and especially upon the women. So much is this the case that bootmakers are loth to construct a square-toed boot at all, and in fashionable shopping districts it is almost impossible to procure such a thing. There is, however, as the London County Council Education Officer points out in a recent report, some mitigation of the evil to be found in that the soft leather of expensive boots and shoes will in time conform itself to the shape of the foot, whereby deformity is at least minimised.

### THE BOOTS OF SCHOOL CHILDREN.

It appears from the same report that the vanity of parents who are forced by circumstances to buy cheap boots for their children impels them to select "fashionable shapes"; and the manufacturers play up to this trait (as indeed they must to succeed in trade competition) by reproducing the pointed toe of the society lady. In such boots the leather, instead of being thin and soft, is hard and thick, and is rendered even more rigid by the nails and rivets by which it is secured. Thus the growing foot is cramped so forcibly that flat foot and other deformities are almost inevitable. In such boots, too, laced tightly up to a point well above the ankle, the movements of that joint are practically inhibited, and natural free movement becomes impossible. An ungainly shuffle is the consequence; and the excessive weight of the boots is another contributing factor which encourages the same tendency. When gymnastic drills and exercises are adopted by children thus crippled their value is entirely lost; and in some schools it has been found advantageous to drill the children in their stockinged feet as the only way of enabling them to profit by the exercises. And this is the twentieth century!

### THIS WEEK'S DRUG MARKET.

At last business in the drug market shows distinct signs of improvement. Large transactions in quinine have been effected at the advanced price, and there has been a good inquiry for a number of other drugs. It is not improbable that a further advance in the price of quinine will take place before long. Cod-liver oil does not seem likely to recede in value again; in fact, there is a slight upward tendency in the price of this oil, and the present is a good time to make purchases. Oil of cloves is dearer in consequence of the increased value of cloves. Thymol, balsam tolu, jalap, buchu leaves, balsam copaiba, and lactic acid are all tending upwards in price.

